# Joint Aperture and Power Allocation Strategy for a Radar Network Localization System Based on Low Probability of Interception Optimization

**DOI:** 10.3390/s23052613

**Published:** 2023-02-27

**Authors:** Chenyan Xue, Ling Wang, Daiyin Zhu

**Affiliations:** 1 Key Laboratory of Radar Imaging and Microwave Photonics, Ministry of Education, Nanjing University of Aeronautics and Astronautics, Nanjing 211106, China; 2 Leihua Electronic Technology Research Institute, Aviation Industry Corporation of China, Wuxi 214063, China

**Keywords:** Distributed Radar Network Localization System (DRNLS), Schleher Interception Factor, Low Probability of Intercept (LPI), Chance Constrained Programmin (CCP), Aperture Resource Allocation (ARA), Radar Cross Section (RCS)

## Abstract

In the process of using the Distributed Radar Network Localization System (DRNLS) further to improve the survivability of a carrier platform, the random characteristics of the system’s Aperture Resource Allocation (ARA) and Radar Cross Section (RCS) are often not fully considered. However, the random characteristics of the system’s ARA and RCS will affect the power resource allocation of the DRNLS to a certain extent, and the allocation result is an essential factor determining the performance of the DRNLS’s Low Probability of Intercept (LPI). Therefore, a DRNLS still has some limitations in practical application. In order to solve this problem, a joint allocation scheme of aperture and power for the DRNLS based on LPI optimization (JA scheme) is proposed. In the JA scheme, the fuzzy random Chance Constrained Programmin model for radar antenna aperture resource management (RAARM-FRCCP model) can minimize the number of elements under the given pattern parameters. The random Chance Constrained Programmin model for minimizing Schleher Intercept Factor (MSIF-RCCP model) built on this basis can be used to achieve DRNLS optimal control of LPI performance on the premise of ensuring system tracking performance requirements. The results show that when RCS has some randomness, its corresponding uniform power distribution result is not necessarily the optimal scheme. Under the condition of meeting the same tracking performance, the required number of elements and power will be reduced to a certain extent compared with the number of elements in the whole array and the power corresponding to the uniform distribution. The lower the confidence level is, the more times the threshold is allowed to pass, and the lower the power is, so that the DRNLS can have better LPI performance.

## 1. Introduction

At present, radar is developing rapidly in the direction of multifunction, digitalization, and integration. In this context, the development cycle of new radar is becoming longer and longer, and it is difficult for new radar to meet the requirements of system performance in technology fully. To effectively solve this problem, the concept of a Distributed Radar Network Localization System (DRNLS) came into being. The DRNLS can make up for the above shortcomings and form a comprehensive, three-dimensional, and multilevel system, significantly improving the system performance, which scholars favor. Since the Low Probability of Interception (LPI) performance is an essential factor affecting the survivability of radar, it is of great theoretical and practical significance to use a DRNLS to allocate power reasonably on the premise of ensuring that the DRNLS meets the LPI performance.

Generally, the influence of the radar network, aperture, and RCS uncertainty on power allocation is the three main factors determining radar LPI performance. In recent studies, deep neural networks have been widely used in natural language processing [1,2], heterogeneous relational attention networks are used to embed learning knowledge maps and computer vision [3,4], and some scholars have studied DRNLS resource allocation. For example, Refs. [5,6,7,8,9,10,11] studied the problems related to a radar network. Refs. [12,13,14,15,16] studied various problems related to allocating radar aperture resources. In Refs. [17,18,19,20,21], relevant scholars studied the allocation of radar power resources.

A radar network is important for improving a radar systems’ detection and LPI performance. Each radar node in a radar network can transmit independent quadrature waveforms (to avoid interference) and receive and process all transmitted waveforms simultaneously [5]. In 2010, Godrich and others [6] studied the impact of different distributed MIMO radar networks on target tracking performance. The research shows that higher target tracking accuracy can be obtained by increasing the number of radar transmitters and receivers and illuminating the target from multiple perspectives. In 2013, Hachour et al. [7] proposed a multisensor multitarget joint tracking and classification algorithm based on creed classification. According to the target motion state and acceleration information, the creed classifier was used to obtain the type set of the target. In 2015, Yang et al. [8] studied the target tracking performance of radar network systems under deception jamming and analyzed the impact of different system parameters on target tracking performance. Sobhani et al. [9] proposed a particle filter algorithm for UWB radar network multitarget tracking. In 2016, Liu et al. [10] proposed a coordinated track initiation algorithm for a radar network system based on the target tracking information, which improved the target track initiation probability of the system. In 2020, Yan et al. [11] proposed a cooperative detection and power allocation strategy for radar network target tracking, which optimizes the false alarm rate and transmission power of each radar node under the constraint of some resource budgets.

DRNLS power resource allocation is an important way to improve LPI performance, including optimizing the peak side lobe level (PSL) of the aperture, the number of elements in the radar aperture, and the power of a single element. Because of the limitation of the traditional radar concept, the traditional pattern synthesis research does not involve the resource management of antenna aperture and the uncertainty of the radar system and target environment. In 1990, Olen et al. [12] proposed an adaptive weight selection algorithm for a given array element set to meet specific side lobe criteria. However, this is only to optimize the weights with ten uniformly distributed elements. Compared with [12], Zhou and Ingram [13] proposed a new adaptive pattern synthesis algorithm in 1999, which can more easily control the main lobe shape of any array. In 2005, Shi et al. [14] proposed a new array pattern synthesis algorithm based on the two-step least squares method. They closed the array pattern by jointly modifying the phase of the desired pattern and the weight vector of the composite pattern. In addition, by simultaneously optimizing the sensor location and array complex weight coefficient to minimize PSLL and in order to maintain the desired beam pattern, in 2011, Cen et al. [15] proposed an improved genetic algorithm for beam pattern synthesis of linear aperiodic arrays with arbitrary geometry. Considering the uncertainty of the radar system and target environment, Gong et al. [16] introduced uncertainty in managing aperture resources in 2014. An optimal objective function can be obtained without all elements being in a working state. However, due to the uncertainty of the radar system and target environment, the aperture length and array element number that determines the pattern quality are uncertain.

Several scholars have studied the problem of DRNLS power resource allocation [17,18,19,20,21], aiming to enable the DRNLS to dynamically coordinate the transmission parameters of all radars and improve the utilization efficiency of resources. Godrich et al. [17] proposed a performance-based power allocation algorithm on the platform of distributed multiple input multiple outputs (MIMO) radars. The main idea of this algorithm is to make the DRNLS consume the least transmit power under the condition of achieving preset localization accuracy. Yan et al. [18] applied the idea of power allocation to 3D target tracking and proposed a cognitive DRNLS target tracking algorithm. However, these algorithms assume that the target’s Radar Cross Section (RCS) information is known before. However, in the actual target location, the RCS information of the target at the next time cannot be obtained at the current time because it is related not only to the type, attitude, and position of the target but also to the angle of view, polarization, incident wavelength, and other factors [22]. In this case, Chavali et al. [19] proposed an antenna selection and power allocation algorithm for multitarget tracking. The algorithm adds the target RCS to the state variable to be estimated and sets its transition model as a first-order Markov process. By recursion of state variables, the RCS of the target at the next time can be predicted at the current time. Then, the BCRLB at the next time can be calculated iteratively and used as the cost function of power allocation. Liu et al. [20] proposed an access control and power allocation algorithm based on CCP by establishing time-varying channels as random variables under the application background of cognitive radio. Yan et al. [21] proposed a DRNLS robust power allocation algorithm based on nonlinear CCP (NCCP) for the random factor of RCS in target tracking. The purpose is to enable the DRNLS to dynamically coordinate the transmission parameters of all radars, thereby saving power resources as much as possible under the condition of meeting opportunity constraints.

In solving optimization problems, Liu et al. [23,24] used the data generated by fuzzy random simulation or random simulation to train a neural network and combined it with a genetic algorithm to form a hybrid intelligent algorithm. Han et al. [25] combined the fuzzy random simulation method with the nondominated sorting genetic algorithm (NSGA) to form a hybrid intelligent optimization algorithm for the fuzzy random Chance Constrained Programmin model of aperture resource management of the antenna array. Compared with the genetic algorithm in Refs. [23,24], the hybrid intelligent optimization algorithm can obtain the optimal solution of the optimization problem without relying on the training of the neural network and can reduce the amount of computation. Therefore, based on the given constraints, it can find the number and layout of array elements that make the objective function optimal. In addition, Godrich et al. [26] creatively proposed an iterative algorithm for the local search of minimum value, which can bridge the nonconvex optimization problem and the corresponding relaxed convex optimization problem. This algorithm can ensure that the optimal solution of the unrelaxed convex optimization problem can be further searched after using CVX to find the optimal solution of the relaxed convex optimization problem.

By combing the above documents, it was found that there are references that analyze how a certain factor in radar netting, aperture, and power affects LPI performance separately, but there is no research that comprehensively considers how these three factors affect LPI performance together. At the same time, in the aspect of solving optimization problems, there is no literature that combines a genetic algorithm to solve the optimal value without training the neural network with the iterative optimization algorithm (IOA) to ensure that the real optimal solution can be found smoothly with moderate calculation. Therefore, this paper proposes a joint allocation scheme of aperture and power for the DRNLS based on LPI (JA scheme). In this scheme, on the one hand, considering the fuzziness and randomness of the array element distribution, the fuzzy random Chance Constrained Programmin model is used to model the aperture optimization problem. On the other hand, due to the randomness of RCS, the stochastic-constrained programming model is used to model the power optimization problem. At the same time, because of the nonconvex nature of most optimization problems, based on properly expanding the IOA, the JA scheme can ensure that the optimal solution of the optimization problem can be found, and it can improve the LPI performance of the DRNLS. In addition, the optimal solution of the aperture optimization problem as one of the initial conditions of the power optimization problem is the key to realizing the joint allocation of aperture and power.

The content of this paper is organized as follows. Section 2 introduces the system model and the comprehensive content of the pattern. Section 3 analyzes the Schleher Interception Factor of the DRNLS. Section 4 establishes the fuzzy random Chance Constrained Programmin model for radar antenna aperture resource management (RAARM-FRCCP model) and the random Chance Constrained Programmin model for minimizing Schleher Intercept Factor (MSIF-RCCP model) to minimize the number of array elements and the system’s Schleher Interception Factor. Section 5 describes the fuzzy random simulation technology with genetic algorithm (FRS-GA) of the RAARM-FRCCP model and the random simulation technology with genetic algorithm (RS-GA) and iterative algorithm for locally searching the minimum (LSMIA) of the MSIF-RCCP model. Section 6 conducts numerical simulations. In this section, the LPI performance of the DRNLS is analyzed first. Then, the resource consumption of the antenna array is minimized under the condition of meeting the chance constraints of the desired pattern parameters. Finally, when the MSE is given, the power allocation strategy with random RCS is compared with the uniform power allocation, and the low intercept performance at different confidence levels is analyzed.

## 2. System Model and Preliminaries

### 2.1. System Model

Consider a DRNLS with *M* transmitting radars and *N* receiving radars. Suppose the DRNLS tracks a target whose position is at (x,y) in the coordinate. The transmitting and the receiving radars are distributed in the same two-dimensional space, and the positions of each radar are arbitrarily distributed. The coordinates of the *M* transmitters and the *N* receivers can be denoted as $(x_{mT},y_{mT}),m=1,\cdots ,M$ and $(x_{nR},y_{nR}),n=1,\cdots ,N$, respectively.

Let $\tau_{m,n}$ represent the total time used for transmitting the signal from the *m*th radar and reflecting it through the target until it is received by the radar *n*, which is also called the signal propagation time, and its expression is

(1) $$\begin{array}{c} \tau_{m,n}=\frac{D_{mT}+D_{nR}}{c} \end{array}$$

where *c* is the speed of light, $D_{mT}$ represents the distance between Transmitter *m* and the target, and $D_{nR}$ represents the distance between Receiver *n* and the target. The calculation formulas of $D_{mT}$ and $D_{nR}$ ares as follows

(2) $$\begin{array}{c} D_{mT}=\sqrt{{\left(x_{mT}-x\right)}^{2}+{\left(y_{mT}-y\right)}^{2}} \end{array}$$

(3) $$\begin{array}{c} D_{nR}=\sqrt{{\left(x_{mR}-x\right)}^{2}+{\left(y_{mR}-y\right)}^{2}} \end{array}$$

According to the definition of $\tau_{m,n}$, the baseband signal of the $\left(m,n\right)$th channel composed of transmitter *m* and receiver *n* can be expressed as

(4) $$\begin{array}{c} r_{m,n}\left(t\right)=\sqrt{\alpha_{m,n}p_{mt}}h_{m,n}s_{m}\left(t-\tau_{m,n}\right)+\omega_{m,n}\left(t\right) \end{array}$$

where $\alpha_{m,n}$ represents the impact of path propagation loss on signal strength, $h_{m,n}$ represents the random variable RCS obeying exponential distribution in the Swerling I model, and $\omega_{m,n}\left(t\right)$ represents the complex white Gaussian noise with circular symmetry and zero mean value. Its autocorrelation function is $\sigma_{\omega }^{2}\delta \left(\tau \right)$, and $p_{mt}$ is the power of the *m*th transmitter.

### 2.2. Pattern Synthesis

Assuming that there are *S* antenna elements with a known distribution, $d_{i}$ indicates the working state of the *i*th array element: one indicates that the array element is in the open state, and zero indicates that the array element is in the closed state. Due to the randomness of the excitation state of the array element and the arbitrariness of the array element position, the number of elements participating in the beam synthesis is uncertain. The decision vector is as follows

(5) $$\begin{array}{c} D={\left[d_{1},d_{2},\cdots ,d_{S}\right]}^{T} \end{array}$$

where $d_{i}=0,1$, and i=1,2,⋯,S.

Suppose that all elements are isotropic, and the coordinates of each element are $\left(x_{i},y_{i},z_{i}\right)$. Thus, the pattern function of the array is

(6) $$\begin{array}{c} p\left(El,Az\right)=\sum_{i=1}^{N}d_{i}I_{i}\exp\left[\frac{j2\pi c\tau_{i}\left(El,Az\right)}{\lambda }\right]\exp\left(j\psi_{i}\right) \end{array}$$

where λ represents the wavelength of the signal; El and Az denote the pitch angle and azimuth angle, respectively; $I_{i}$ represents the current amplitude of the *i*th array element, whose default value is one; and $\psi_{i}$ represents the current phase of the *i*th array element, whose default value is zero. Relative to the phase reference point, the expression of time delay $\tau_{i}\left(El,Az\right)$ of the *i*th array element is

(7) $$\begin{array}{c} \tau_{i}\left(El,Az\right)=\frac{x_{i}\sin El\cos Az+y_{i}\sin El\sin Az+z_{i}\cos El}{c} \end{array}$$

## 3. Schleher Interception Factor Analysis of Radar Network System

Based on the analysis of the Schleher Interception Factor and the relationship between the Schleher Interception Factor and various parameters, various ways to improve the LPI performance of the DRNLS are obtained.

### 3.1. Calculation of Schleher Interception Factor

For a DRNLS, we assume that each radar receiver can distinguish all signals and all antenna beams point to the same target. In addition, suppose that each radar is the same, the transmitter–receiver pairs are formed in the same way, and there is $D_{net}^{2}≜D_{mT}\cdot D_{nR}$. Then, the range equation of the DRNLS can be written as [27]

(8) $$\begin{array}{c} D_{net}^{}={\left[N_{r}\frac{P_{t}G_{t}G_{r}\lambda^{2}\sigma }{SNR_{mon}{\left(4\pi \right)}^{3}P_{rd}L_{rd}}\right]}^{\frac{1}{4}} \end{array}$$

where $P_{t}$ is the total peak power of the DRNLS. The range equation of monostatic radar is

(9) $$\begin{array}{c} D_{mon}^{}={\left[\frac{P_{t}G_{t}G_{r}\lambda^{2}\sigma }{SNR_{mon}{\left(4\pi \right)}^{3}P_{rd}L_{rd}}\right]}^{\frac{1}{4}} \end{array}$$

The range equation of the intercepting receiver is

(10) $$\begin{array}{c} D_{esm}^{}={\left[\frac{P_{t}^{}G_{t,esm}G_{esm}\lambda^{2}}{SNR_{esm}{\left(4\pi \right)}^{2}P_{esm}L_{esm}}\right]}^{\frac{1}{2}} \end{array}$$

The interception receiver can realize the interception detection of its signal by receiving the main lobe or side lobe radiation energy of the radar. According to Equations (8) and (10), the main lobe interception factor $\alpha_{net}$ and side lobe interception factor $\alpha_{net}^{{}^{\prime }}$ of the DRNLS can be calculated as:

(11) $$\begin{array}{c} \alpha_{net}=\frac{D_{esm}}{D_{net}}={\left[\frac{1}{N_{r}}\cdot \frac{\lambda^{2}}{4\pi \sigma }\cdot \frac{P_{t}G_{t,esm}}{G_{r}}\cdot \frac{G_{esm}^{2}P_{rd}SNR_{mon}L_{rd}}{P_{esm}^{2}SNR_{esm}^{2}L_{esm}^{2}}\right]}^{\frac{1}{4}} \end{array}$$

(12) $$\begin{array}{c} \alpha_{net}^{{}^{\prime }}=\frac{D_{esm}}{D_{net}}={\left[\frac{1}{N_{r}}\cdot \frac{\lambda^{2}}{4\pi \sigma }\cdot \frac{P_{t}G_{t,esm}^{2}}{G_{t}G_{r}}\cdot \frac{G_{esm}^{2}P_{rd}SNR_{mon}L_{rd}}{P_{esm}^{2}SNR_{esm}^{2}L_{esm}^{2}}\right]}^{\frac{1}{4}} \end{array}$$

where $N_{r}$ is the number of DRNLS radar receivers, $P_{t}$ is the total peak power of the radar-transmitted signal, $G_{t}$ is the gain of the radar transmitting antenna in the direction of the target, $G_{r}$ is the gain of the radar receiving antenna in the direction of the target, $G_{t,esm}$ is the gain of the radar transmitting antenna in the direction of the intercepting receiver, $G_{esm}$ is the gain of the intercepting receiver antenna, $P_{rd}$ is the sensitivity of the radar receiver, $P_{esm}$ is the sensitivity of the interception receiver, λ is the radar wavelength, σ is the effective scattering area of the radar target, $L_{rd}$ is the radar system loss, $L_{esm}$ is the system loss coefficient from the radar antenna to the interception receiver, $SNR_{mon}$ is the SNR at the input of the signal processor of the monostatic radar receiver, and $SNR_{esm}$ is the SNR that intercepts the input of the receiver signal processor.

### 3.2. Relationship between Schleher Interception Factor and Parameters

#### 3.2.1. Number $N_{r}$ of Radar Network Receivers

According to Equation (9), the maximum detection range of the monostatic radar is

(13) $$\begin{array}{c} D_{mon}^{\max}={\left[\frac{P_{mon}^{\max}}{SNR_{mon}}\cdot \frac{G_{t}G_{r}\lambda^{2}\sigma }{{\left(4\pi \right)}^{3}P_{rd}L_{rd}}\right]}^{\frac{1}{4}} \end{array}$$

where $P_{mon}^{\max}$ is the maximum transmission power of the monostatic radar. Similarly, for the interception receiver, there is the following equation

(14) $$\begin{array}{c} D_{esm}^{\max}={\left[\frac{P_{mon}^{\max}}{SNR_{esm}}\cdot \frac{G_{t,esm}G_{esm}\lambda^{2}}{{\left(4\pi \right)}^{2}P_{esm}L_{esm}}\right]}^{\frac{1}{2}} \end{array}$$

where $D_{esm}^{\max}$ represents the interception distance corresponding to the monostatic radar’s maximum transmission power $P_{mon}^{\max}$. Therefore, by Equations (9), (10), (13), and (14), it can be obtained that

(15) $$\begin{array}{c} \frac{\alpha_{mon}}{\alpha_{mon}^{\max}}=\left.\left(\frac{D_{esm}}{D_{mon}}\right)/\left(\frac{D_{esm}^{\max}}{D_{mon}^{\max}}\right)\right.={\left(\frac{P_{t}}{P_{mon}^{\max}}\right)}^{\frac{1}{4}} \end{array}$$

where $\alpha_{mon}$ is the Schleher Interception Factor of the monostatic radar, and $\alpha_{mon}^{\max}$ is the Schleher Interception Factor corresponding to the maximum transmission power $P_{mon}^{\max}$ of the monostatic radar. To simplify the calculation, when the monostatic radar transmits the full power $P_{mon}^{\max}$, the Schleicher interception factor $\alpha_{mon}^{\max}$ is normalized to one. Thus, when the total transmission power of the radar network system is $P_{t}$, Equation (15) can be simplified as follows

(16) αnet=DesmDmonNr14=αmonNr14=PtPmonmax·Nr14

It can be seen from Equation (16) that with the increase of the number of radar receivers $N_{r}$ in the network and the decrease of the total transmission power $P_{t}$ of the system, the Schleher Interception Factor of the DRNLS also decreases.

#### 3.2.2. Gain $G_{t,esm}$ of the Radar Transmission Antenna in the Direction of the Reconnaissance Receiver

According to Equation (11), the relationship between the interception factor and radar parameters is as follows

(17) $$\begin{array}{c} \alpha_{net}\propto {\left(G_{t,esm}\right)}^{\frac{1}{2}} \end{array}$$

It can be seen from Equation (17) that a lower interception factor can be obtained by reducing the gain of the radar transmitting antenna in the direction of the interception receiver. This gain may be the main lobe gain of the radar antenna or the side lobe gain of the radar antenna. Due to the discrete scanning of the phased array radar, the time for the intercepting receiver to intercept the main lobe of the radar is very short. Therefore, reducing the side lobe of the radar transmitting antenna is one of the effective measures to achieve low interception.

The time for the intercepting receiver to intercept the main lobe of our radar in the airspace is much less than the time for the side lobe. For example, the radar tracks the target, and the target is equipped with a self-defense jammer. The radar tracks the target for only a few tracking frames in a cycle, i.e., the radar’s main lobe illuminates the target, and the radar side lobes illuminate the target at other times. For an intercepting receiver, the probability of signal interception is defined as:

(18) P=1−1−PfaTτ

where $P_{fa}$ is the interception probability in time τ, and *T* is the total interception time. Therefore, suppose that τ is the dwell time of the main lobe illuminating the target, and *T* is an airspace scanning period of the radar. If the low side lobe antenna is adopted, the radar will not be intercepted in time T−τ, and the probability of interception will be significantly reduced. The interception probability under different conditions is shown in Table 1.

#### 3.2.3. Radar Power Gain Product $P_{t}G_{t}$

According to Equation (11), the relationship between the interception factor and the radar parameter is as follows

(19) $$\begin{array}{c} \alpha_{net}\propto {\left(P_{t}G_{t}\right)}^{\frac{1}{4}} \end{array}$$

As shown in Equation (19), low transmission peak power and transmission antenna gain are adopted, and the low interception factor can be effectively reduced by controlling the radar power gain product. From the analysis result of the interception factor, reducing the radar transmission peak power is one of the effective low interception measures. If we reduce the peak power of radar transmission, increase the duty cycle of the radar waveform, and ensure the average power of the radar transmission has no impact on radar detection performance, we can effectively reduce the interception distance of the other party. In practical engineering, when the signal-to-noise ratio of the target echo is too large, i.e., the radar does not need to use too large a peak power gain product, this measure can be taken to reduce the power gain product of the radar peak, reducing the intercepted distance while ensuring the radar detection range.

The above analysis shows that under the condition of a certain radar receiver sensitivity, the methods to improve the radar interception factor include: increasing the number of radar network receivers, reducing the radar transmit power gain product, and reducing the radar transmit antenna side lobe level.

## 4. Joint Optimal Control Algorithm of Aperture and Power

In this section, a JA scheme is proposed to improve the low interception performance of a DRNLS by reducing the number of radar excitation elements and the radiation power of each excitation element in the target location process under the low side lobe constraint. Section 4.1 establishes the RAARM-FRCCP model; Section 4.2 is the CRB of target positioning; in Section 4.3, based on the optimization of the number of elements in Section 4.1, considering the randomness of RCS, the MSIF-RCCP model is established to minimize the system Schleher interception factor.

### 4.1. Establishment of RAARM-FRCCP Model for Aperture Distribution

If all the elements are in the excited state, it will waste antenna array resources. Due to the array elements’ arbitrary distribution and the working state’s uncertainty, the radar system can synthesize beams that meet the requirements of NPSLL and main lobe width when the number of excitation elements is less than SS. However, when the number of array elements participating in pattern synthesis is too small, the far-side lobe level will be very high. Therefore, the number of array elements in the excitation state is distributed in an appropriate interval. The number and location of elements selected each time to participate in pattern synthesis are random. However, due to too many combinations between elements, it is difficult to obtain their random distribution function through repeated experiments. Then, fuzzy random variables can be introduced to represent the number of exciting elements, and the RAARM-FRCCP model can be established [28]. Because of the arbitrary distribution of array element positions and the uncertainty of the working state of the array element, the calculation results sometimes cannot fully achieve the desired optimization goal. Only when the chance measure of the constraint conditions is greater than or equal to the pre-given confidence level can the optimization goal be met.

The optimal number of elements in the working state $\xi \left(\omega \right)$ represents the number of elements in the excited state, where ω is a random number. $P_{NPSLL}\left(D,\xi \right)$ and $B\left(D,\xi \right)$ are the normalized peak side lobe level and the calculated first zero main lobe width, respectively. Establish the following Chance Constrained Programmin model

(20) $$\begin{array}{c} \left\{\begin{array}{cc} \; & \min\;\;\bar{f}\\ s.t. & \mathrm{Ch}\left\{\xi ⩽\bar{f}\right\}\left(\gamma \right)⩾\delta ,\\ \; & \mathrm{Ch}\left\{P_{NPSLL}\left(D,\xi \right)-P_{NPSLL}⩽0\right\}\left(\gamma \right)⩾\beta_{1},\\ \; & \mathrm{Ch}\left\{B\left(D,\xi \right)-B_{W}\right\}\left(\gamma \right)⩾\beta_{2},\\ \; & D={\left[d_{1},d_{2},\cdots d_{S}\right]}^{T},\\ \; & \sum_{i=1}^{S}d_{i}=\xi . \end{array}\right. \end{array}$$

where $P_{NPSLL}$ and $B_{W}$ are the expected normalized NPSLL and the first zero main lobe widths, respectively, assume that γ, δ, $\beta_{1}$, and $\beta_{2}$ are confidence levels. $\xi \left(\omega \right)$ is a fuzzy random variable, and $\bar{f}$ is the threshold of the number of elements in the working state.

### 4.2. CRB for Target Localization

CRB provides a lower bound for mean square error (MSE) of unbiased estimation of unknown parameters. Given a parameter vector u, the component is $u_{i}$, and its unbiased estimate ${\hat{u}}_{i}$ satisfies the following inequality [26]

(21) $$\begin{array}{c} E_{u}\left\{\left(\hat{u}-u\right){\left(\hat{u}-u\right)}^{T}\right\}⩾J^{-1}\left(u\right) \end{array}$$

where parameter vector u

(22) $$\begin{array}{c} u={\left[x,y,h^{T}\right]}^{T} \end{array}$$

In the above formula, $h={\left[h_{1,1},h_{1,2},\cdots ,h_{M,N}\right]}^{T}$ represents the target RCS observed by different receiving and transmitting paths.

In Equation (21), $J\left(u\right)$ is the Fisher information matrix (FIM), which has the following formula [26]

(23) $$\begin{array}{c} J\left(u\right)=E_{\left.r\right|u}\left\{\frac{\partial }{\partial u}\log f\left(r|u\right){\left(\frac{\partial }{\partial u}\log f\left(r|u\right)\right)}^{T}\right\} \end{array}$$

where $f\left(r|u\right)$ is the condition of observation vector $r={\left[r_{1,1},r_{1,2},\cdots ,r_{M,N}\right]}^{T}$ and the joint probability density function. As an exponential random variable, the target RCS is more consistent with the real situation than a deterministic variable. Meanwhile, Equation (4) shows that the target RCS with randomness affects the baseband distribution to a certain extent. See Appendix A for $f\left(r|u\right)$ conditional probability density function.

CRB matrix $C_{x,y}$, defined as the 2×2 matrix of the upper left block of FIM’s inverse matrix $J^{-1}\left(u\right)$, is expressed by the following matrix [29]

(24) $$\begin{array}{c} C_{x,y}\left(u,p_{t}\right)={\left\{\sum_{m=1}^{M}p_{mt}\left[\begin{array}{cc} \Xi_{am} & \Xi_{cm}\\ \Xi_{cm} & \Xi_{bm} \end{array}\right]\right\}}^{-1} \end{array}$$

The component elements $\Xi_{am}$, $\Xi_{bm}$. and $\Xi_{cm}$ are, respectively, defined as [26]

(25) $$\begin{array}{c} \Xi_{am}=\xi_{m}\sum_{n=1}^{N}\alpha_{m}{\left|h_{m,n}\right|}^{2}\left(\frac{x_{mT}-x}{D_{mT}}+\frac{x_{nR}-y}{D_{nR}}\right) \end{array}$$

(26) $$\begin{array}{c} \Xi_{bm}=\xi_{m}\sum_{n=1}^{N}\alpha_{m}{\left|h_{m,n}\right|}^{2}\left(\frac{y_{mT}-x}{D_{mT}}+\frac{y_{nR}-y}{D_{nT}}\right) \end{array}$$

(27) $$\begin{array}{c} \Xi_{cm}=\xi_{m}\sum_{n=1}^{N}\alpha_{m}{\left|h_{m,n}\right|}^{2}\left(\frac{x_{mT}-x}{D_{mT}}+\frac{x_{nR}-y}{D_{nT}}\right)\left(\frac{y_{mT}-x}{D_{mT}}+\frac{y_{nR}-y}{D_{nT}}\right) \end{array}$$

Among them, ξm=8π2(βm2σw2c2). $\beta_{m}$ is the effective bandwidth of the *m*th transmitting radar. The elements of the CRB matrix depend on the azimuth of the transmitting and receiving antennas relative to the target (taking the *x* axis as the standard). The trace of the $C_{x,y}$ matrix represents the lower bound of the MSE sum estimated in all directions of the target position, such as in the two-dimensional plane, $tr\left(C_{x,y}\right)⩽\sigma_{x}^{2}+\sigma_{y}^{2}$, where $\sigma_{x}^{2}$ and $\sigma_{y}^{2}$ represent the MSE estimated in the *x* and *y* axes, respectively. After a series of matrix calculations, the trace of the CRB matrix $C_{x,y}$ can be expressed as [26]

(28) $$\begin{array}{c} \sigma_{x,y}^{2}\left(p_{t}\right)=\mathrm{tr}\left(C_{x,y}\right)=\frac{b^{T}p_{t}}{p_{t}^{T}Ap_{t}} \end{array}$$

Among them, $p_{t}={\left[p_{1t},p_{2t},\cdots ,p_{Mt}\right]}^{T}$, $b=\left(\Xi_{a}+\Xi_{b}\right)$, $A=\Xi_{a}\Xi_{b}^{T}-\Xi_{c}\Xi_{c}^{T}$. The component elements in $\Xi_{a}={\left[\Xi_{a1},\Xi_{a2},\cdots ,\Xi_{aM}\right]}^{T}$, $\Xi_{b}={\left[\Xi_{b1},\Xi_{b2},\cdots ,\Xi_{bM}\right]}^{T}$, and $\Xi_{c}={\left[\Xi_{c1},\Xi_{c2},\cdots ,\Xi_{cM}\right]}^{T}$ vectors are defined in Equations (25), (26) and (27), respectively.

### 4.3. MSIF-RCCP Model Construction for Minimizing Schleher Interception Factor

The relationship between the transmission power and the number of elements is $p_{mt}=p_{mt}^{{}^{\prime }}\cdot \xi^{*}$, where $\xi^{*}$ is the optimal number of elements obtained in Section 4.2, $p_{mt}^{{}^{\prime }}$ is the power of a single element, and *m* is the transmitter number. The above influencing factors are included in the CRB matrix defined by Equation (28) through vectors b, $p_{t}$, and A. The transmit power of each radar is limited between $p_{mt\min}$ and $p_{mt\max}$.

This section will introduce how to allocate the power of each transmitter to minimize the Schleher Interception Factor of the system under the condition of given MSE, $\eta_{\max}$ of the target location. It can be summarized as follows

(29) $$\begin{array}{c} \left\{\begin{array}{ccc} \; & \min_{p_{t}}\;\alpha_{\mathrm{net}} & \\ s.t. & p_{mt}-p_{mt\max}⩽0, & m=1,\cdots ,M,\\ \; & p_{mt\min}-p_{mt}⩽0, & m=1,\cdots ,M,\\ \; & tr\left(C_{x,y}\left(\tilde{u},p_{t}\right)\right)=\eta_{\max}. & \end{array}\right. \end{array}$$

where $C_{x,y}\left(\tilde{u},p_{t}\right)$ is the CRB matrix of 2×2 defined in Equation (28), and $\tilde{u}={\left[\tilde{x},\tilde{y},{\tilde{h}}^{T}\right]}^{T}$ is the prior estimation of the target position and RCS obtained through previous multiple observations.

For each power vector $p_{t}$, the small increment $0<△p⩽p_{mt}$ of transmission power will cause MSE to decrease [26]. Therefore, Equation (29) can be rewritten as follows

(30) $$\begin{array}{c} \left\{\begin{array}{ccc} \; & \min_{p_{t}}\;\alpha_{\mathrm{net}} & \\ s.t. & p_{mt}-p_{mt\max}⩽0, & m=1,\cdots ,M,\\ \; & p_{mt\min}-p_{mt}⩽0, & m=1,\cdots ,M,\\ \; & \eta_{\max}p_{t}^{T}A_{e}p_{t}-b_{e}^{T}p_{t}=0. & \end{array}\right. \end{array}$$

The vector $b_{e}=b\left(\tilde{u}\right)$ and matrix $A_{e}=A\left(\tilde{u}\right)$ are calculated using the estimation vector $\tilde{u}$. The optimization problem of Equation (30) is nonconvex because the third constraint in Equation (30) is an equality constraint [30].

Solving constrained, nonlinear, and nonconvex optimization problems is a challenging task that usually requires much computation. The common solution to such problems is to relax the original problem convexly and then find a local minimum [31] with the optimal solution. The constraint relaxation and power allocation algorithm will be described in detail below.

In order to solve the optimization problem in Equation (30), the third equation $\eta_{\max}p_{t}^{T}A_{e}p_{t}-b_{e}^{T}p_{t}=0$ in the problem can be relaxed first. Since there is transmission power $p_{mt}\neq 0$ for ∀m=1,2,⋯,M, the third equation constraint can be replaced by $\eta_{\max}A_{e}p_{t}-b_{e}=0$. The gradual reduction of transmission power will make MSE gradually larger. After multiple iterations, MSE will be infinitely close to the threshold value $\eta_{\max}$ of the given positioning error. Then, the equality constraint $\eta_{\max}A_{e}p_{t}-b_{e}=0$ can be replaced by the inequality constraint $p_{t}^{T}\left(b_{e}-\eta_{\max}A_{e}p_{t}\right)⩽0$. The relaxed convex optimization problem is given below

(31) $$\begin{array}{c} \left\{\begin{array}{ccc} \; & \min_{p_{t}}\;\alpha_{\mathrm{net}} & \\ s.t. & p_{mt}-p_{mt\max}⩽0, & m=1,\cdots ,M,\\ \; & p_{mt\min}-p_{mt}⩽0, & m=1,\cdots ,M,\\ \; & b_{e}-\eta_{\max}A_{e}p_{t}⩽0. & \end{array}\right. \end{array}$$

In practical applications, target RCS is related to target recognition, attitude, and position and is also affected by azimuth, wavelength, polarization, and other factors [32], which are unknown and uncertain. Therefore, this paper considers the target RCS a random variable. Therefore, the deterministic resource allocation model cannot reflect the characteristics of the target well and truly. Because of the above situation, the random CCP method of resource management is introduced [24]. Therefore, according to Equation (31), the MSIF-RCCP model can be expressed as

(32) $$\begin{array}{c} \left\{\begin{array}{ccc} \; & \min_{p_{t}}\;\alpha_{\mathrm{net}} & \\ s.t. & p_{mt}-p_{mt\max}⩽0, & m=1,\cdots ,M,\\ \; & p_{mt\min}-p_{mt}⩽0, & m=1,\cdots ,M,\\ \; & \mathrm{Pr}\left(b_{e}-\eta_{\max}A_{e}p_{t}⩽0\right)⩾\alpha . & \end{array}\right. \end{array}$$

The probability symbol $\mathrm{Pr}\left(\cdot \right)$ is obtained from the random variable $h_{m,n}$, and α represents the confidence level.

## 5. Joint Optimal Control Algorithm of Aperture and Power

For the fuzzy random Chance Constrained Programmin model of array aperture resource management, the fuzzy random simulation can solve the constraint interval of the number of array elements satisfying the confidence level and then combined with the genetic algorithm to form a hybrid intelligent algorithm (FRS-GA), so that the optimal number and arrangement of array elements can be found under the optimal NPSLL constraints [25]. The random simulation algorithm [28] is embedded into the genetic algorithm to form a hybrid intelligent algorithm (RS-GA) so that the optimal solution $p_{t,opt}^{*}$ can be obtained by solving the relaxed convex optimization problem in Equation (29) under the constraint condition of meeting the given tracking error.

For the optimization process, the detailed steps are shown in Algorithm 1.

**Algorithm 1** Hybrid Intelligent Algorithm

 **Require:**
The population size pop_size, the initialized chromosome set $V=\{d_{1},d_{2},\cdots ,d_{S}\}$, theprobability $P_{c}$ and $P_{m}$ of crossover and mutation, and the number of evolution iterations *N*.
 **Ensure:**
The optimal number of array elements $\xi^{*}$ and minimum LPI $\alpha_{net}^{*}$.1:
**function** FRS-GA Algorithm
2: **Screen out** the feasible initial chromosomes from the set V using the fuzzy random simulation method, and denote the set composed of all the screened initial chromosomes as $V_{1}$;3: **Verify** the feasibility of the chromosomes in the set $V_{1}$ through the fuzzy random simulation method, correct the unfeasible chromosomes, and denote the corrected new set as $V_{2}$;4: **Calculate** the objective function value of all chromosomes in the set $V_{2}$ and further calculate the fitness function value of each chromosome from the obtained objective function value;5: **Select** chromosomes by the roulette method;6: **Repeat** Step3–Step5 until the given number *N* of evolution iterations is met;7: **Take** the best chromosome as the optimal number of array elements $\xi^{*}$ for the optimization problem (20) and return;8:
**end function**
9: 10:
**function** RS-GA Algorithm
11: **Initialize** the population size pop_size and the chromosome set $V_{3}=\left\{p_{1t},p_{2t},\cdots ,p_{Mt}\right\}$ according to the relationship $p_{mt}=p_{mt}^{{}^{\prime }}\cdot \xi^{*}$ and the optimal number of array elements $\xi^{*}$ in the FRS-GA algorithm;12: **Screen out** the feasible initial chromosomes from the set $V_{3}$ using the random simulation method, and denote the set composed of all the screened initial chromosomes as $V_{4}$;13: **Verify** the feasibility of the chromosomes in the set $V_{4}$ through the random simulation method, correct the unfeasible chromosomes, and denote the corrected new set as $V_{5}$;14: **Calculate** the objective function value of all chromosomes in the set $V_{5}$ and further calculate the fitness function value of each chromosome from the obtained objective function value;15: **Select** chromosomes by the roulette method;16: **Repeat** Step13–Step15 until the given number *N* of evolution iterations is met;17: **Take** the best chromosome as the globally optimal power $p_{t,\mathrm{opt}}^{*}$ for the relaxed optimization problem (32) and return;18:
**end function**
19: 20:
**function** LSMIA Algorithm
21: **Initialize** iteration step size $\Delta p_{0}$ and termination condition ε;22: **Assign** $p_{t,\mathrm{opt}}^{*}$ to the starting point $p_{t_{0}}$ to calculate $\sigma_{x,y}^{2}\left(p_{t_{0}}\right)$ in Equation (28);23: **if** $\;\mathrm{Pr}(|\eta_{\max}-\sigma_{x,y}^{2}\left(p_{t(k-1)}\right)|<\varepsilon )⩾\alpha$ **then**24:   **repeat** the following procedure:25:

$\;\;p_{t(k)}=\arg\min\sigma_{x,y}^{2}\left(p_{t(k-1)}\right)\left|<\varepsilon \right)-\eta_{\max}$

 

$\;\;\;s.t.\;p_{mt,\min}⩽p_{mt(k-1)}$

 

$\;\;\;\Delta p_{\left(k\right)}=\Delta p_{\left(k-1\right)}\left[1^{T}p_{t(k)}/1^{T}p_{t(k-1)}\right]$

26: **else** $\;\mathrm{Pr}(|\eta_{\max}-\sigma_{x,y}^{2}\left(p_{t(k-1)}\right)|<\varepsilon )<\alpha$27: **Let** $p_{t,\mathrm{opt}}=p_{t(k-1)}$ be the globally optimal power for the optimization problem (30);28: **end if**29: **Substitute** $p_{t,\mathrm{opt}}$ into Equation (16) to obtain the minimum system interception factor $\alpha_{net}^{*}$.30:
**end function**



Based on the above hybrid intelligent algorithm, the corresponding RAARM-FRCCP model and MSIF-RCCP model can be simulated and analyzed in the next section.

## 6. Numerical Simulation

This section will verify and analyze the JA scheme proposed above through simulation experiments. The experimental scenario is a $5\times 1\left(M=5,N=1\right)$ distributed radar system layout. The antenna of each radar is a square with a side length of 27λ, and 784 array elements are scattered inside. In order to analyze the impact of the power optimization algorithm in the JA scheme on radar LPI performance, this paper uses fixed power radar assignment (FPRA) for performance comparison. In FPRA mode, the power of a single radar to illuminate the target is fixed at 40 kW. The hardware of the experiment is a high-performance computer.

Refs. [23,24] point out that the genetic algorithm is very robust to the parameter setting of population size, crossover probability, and mutation probability, and changing these parameters will have little impact on the results. Therefore, this section sets the population size of the FRS-GA algorithm and the RS-GA algorithm to 100, the crossover probability and mutation probability are $P_{c}=0.9$ and $P_{m}=0.1$, respectively, and the genetic algebra is 300. The number of fuzzy random simulations and random simulations is 1000.

As for the statistical characteristics of the Radar Cross Section (RCS) of aircraft targets, when the aircraft flies in more curves and the flight course changes more frequently, the radar will measure the RCS of the aircraft in more directions, and then the RCS can be regarded as a random process under different attitudes. Under the condition that the number of measurements is large enough and the aircraft attitude changes are large enough, the situations involved are richer and more random [33]. According to the central limit theorem, the RCS of the aircraft is a Gaussian process; in theory, that is, the echo amplitude follows the Rayleigh distribution, and the RCS follows an exponential distribution. In practical application, the independent variable usually takes value in a reasonably limited range, and many theoretical values of probability distributions tend to be positive or negative infinity. Therefore, if the distribution interval is truncated properly, the theoretical analysis results can be more consistent with the actual situation. See Appendix B for the certification process.

**Figure 1:**
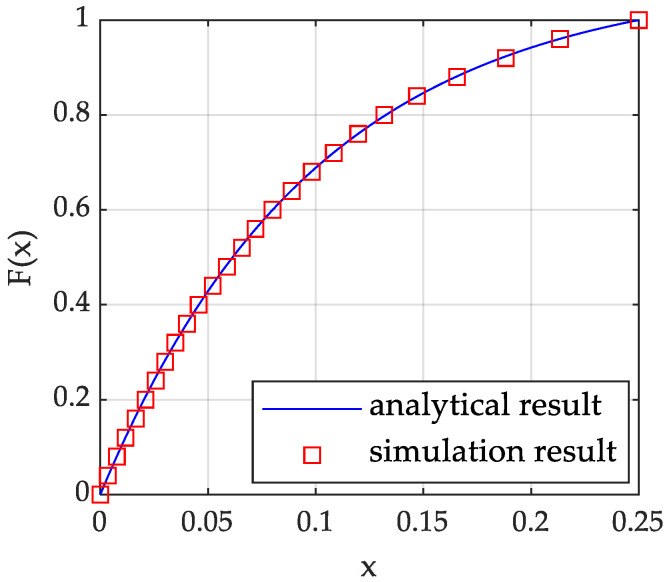
CDF of RCS model ${\left|h_{11}\right|}^{2}$.

**Figure 2:**
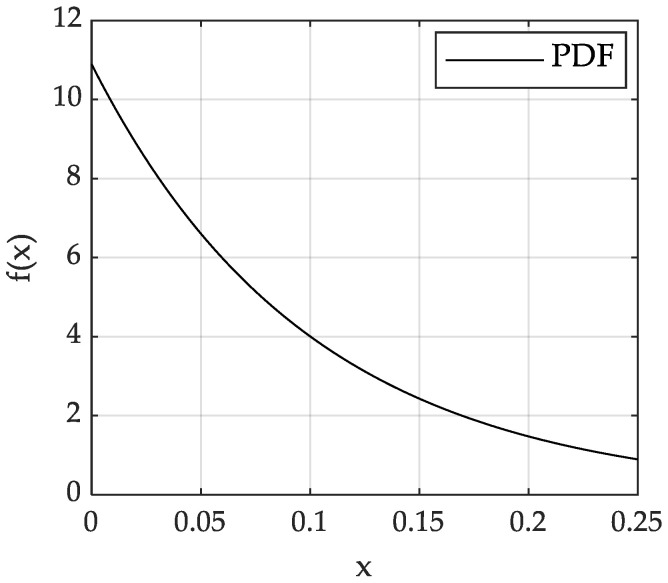
PDF of RCS model ${\left|h_{11}\right|}^{2}$.

Considering that the RCS of each transceiver path can be modelled as a random variable subject to the Swerling I distribution, the RCS of five different transceiver paths is ${\left|h\right|}^{2}=\left[{\left|h_{11}\right|}^{2},{\left|h_{12}\right|}^{2},{\left|h_{13}\right|}^{2},{\left|h_{14}\right|}^{2},{\left|h_{15}\right|}^{2}\right]$, and their mean $h_{av}$ is 0.1,1,0.5,1,0.1, respectively. After the RCS is truncated, it still obeys the Swerling I distribution. Take ${\left|h_{11}\right|}^{2}$ as an example. Let ${\left|h_{11}\right|}^{2}$ be *x*, the cumulative distribution function (CDF) be $F\left(x\right)$, the probability density function (PDF) be $f\left(x\right)$, and the truncation interval is $\left[0,0.25\right]$. Figure 1 shows the comparison of theoretical and analog CDF values of ${\left|h_{11}\right|}^{2}$. Figure 2 shows that the PDF form of ${\left|h_{11}\right|}^{2}$ is still an exponential distribution. It can be seen that the theoretical and analog values are consistent.

### 6.1. Low Interception Performance of Radar Network

In order to verify the feasibility and effectiveness of the radar network target location based on low interception performance optimization, this section gives the relationship curve between the CRB (Equation (28)) and the interception factor (Equation (16)) under different radar network structures. According to Equation (28), ${\sigma_{x,y}}^{2}\left(p_{t}\right)$ is a function of the RCS random variable, so it is still a random variable. According to the probability theory, there are essential differences between the calculation methods of ${\sigma_{x,y}}^{2}\left(p_{t}\right)$ with randomness and ${\sigma_{x,y}}^{2}\left(p_{t}\right)$ with certainty. On the one hand, considering the complexity of Equation (28), it is very difficult to calculate the probability density function of ${\sigma_{x,y}}^{2}\left(p_{t}\right)$. The use of digital features in practical applications is often better than the use of PDF [34]. On the other hand, to obtain the digital features of ${\sigma_{x,y}}^{2}\left(p_{t}\right)$, it is theoretically necessary to know the specific form of its PDF, and the corresponding empirical PDF cannot be directly calculated in the simulation process. However, an empirical CDF can often be obtained directly during the simulation. Therefore, the method of calculating digital features using the CDF is given in Appendix C.

Through observation, it is found that when the interception factor becomes larger in the linear coordinate system, the CRB value tends to zero at a faster rate. This feature makes the CRB values of different cases almost coincide, and it is not easy to distinguish the differences between them. Because of this, this paper intends to use a double logarithmic coordinate system to make the difference between CRB values more obvious in many cases to facilitate observation.

**Figure 3:**
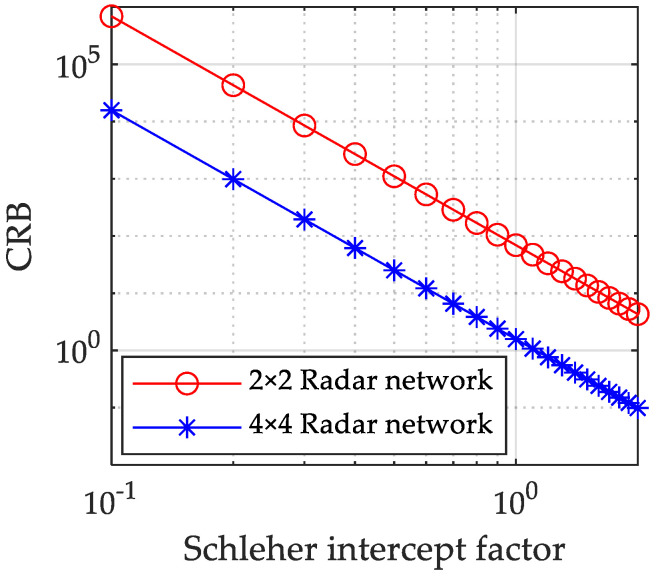
Relation curve between CRB and Schleher Interception Factor under different radar network structures.

It can be seen from Figure 3 that when the Schleher Interception Factor changes from $\alpha_{net}=0$ to $\alpha_{net}=2$, the CRB also decreases. The reason is that when the Schleher Interception Factor increases, the system needs to transmit more power. According to Equation (28), CRB will decrease with the increase of the system transmission power. In addition, it can be seen from Figure 3 that under the same target tracking threshold, the Schleher Interception Factor decreases greatly with the increase in the number of transmitters and receivers in the netted radar system. Therefore, under the same target tracking performance, the increase in the number of radar nodes in the system can effectively improve the low interception performance of the radar network system.

**Figure 4:**
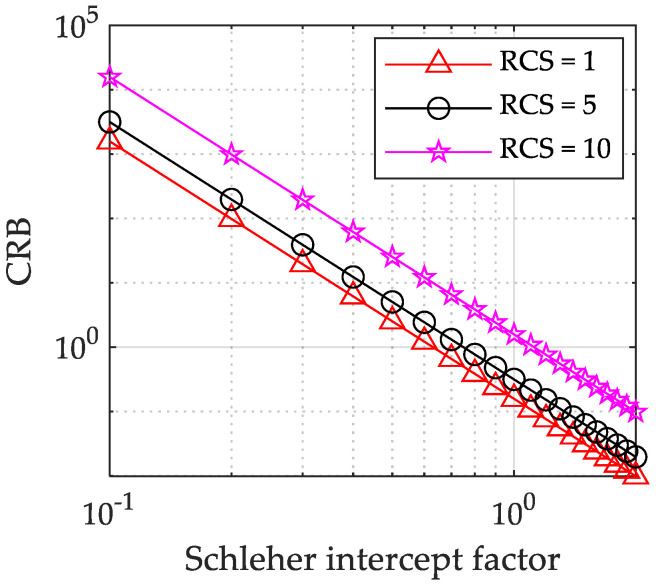
Relation curve between CRB and Schleher Interception Factor under different target RCS strengths (4×4 Radar network).

Figure 4 shows the relationship curve (4×4 Radar network) between CRB and Schleher Interception Factor under different target RCS. As shown in Figure 4, when the target RCS mean $h_{av}$ increases from 1 m${}^{2}$ to 10 m${}^{2}$, the CRLB decreases accordingly. The reason is that the radar network system is easier to locate targets with high scattering intensity in the target location process.

### 6.2. Aperture Assignment

**Figure 5:**
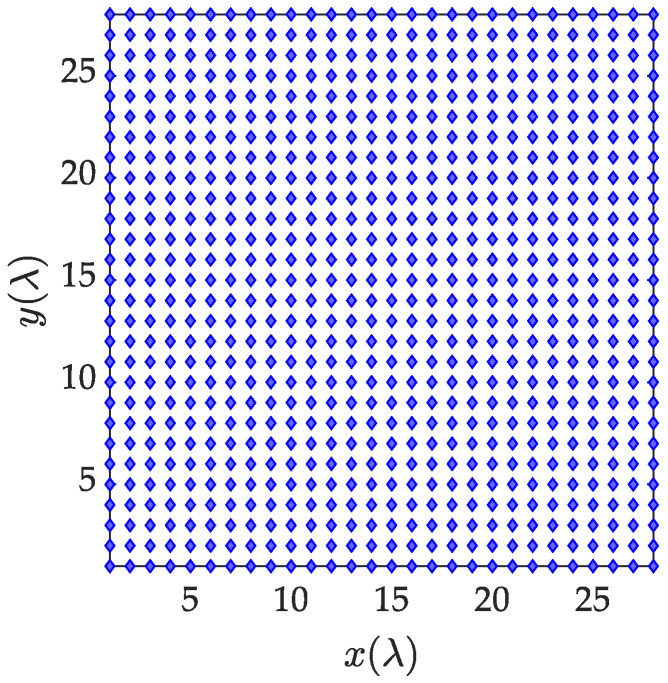
Distribution of array elements before optimization.

**Figure 6:**
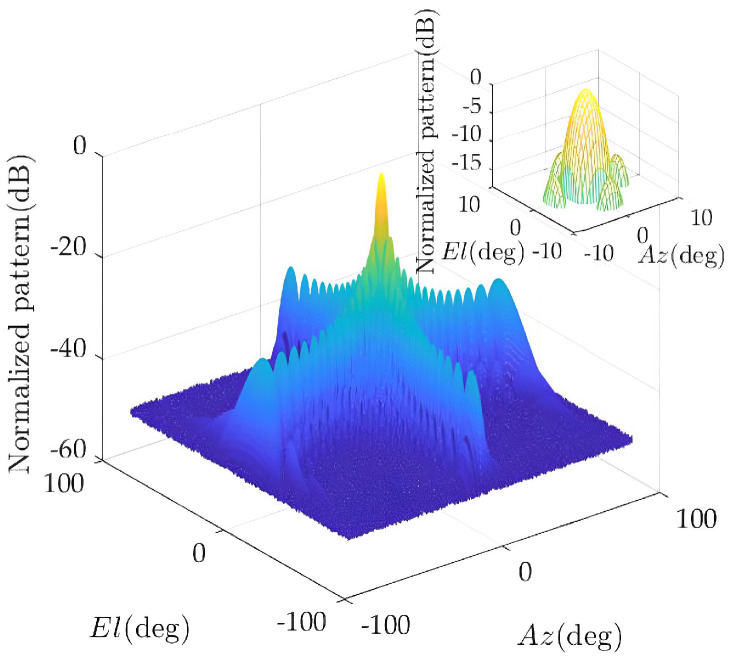
Pattern of antenna array before optimization.

As shown in Figure 5, 784 antenna elements are scattered in the circle with radius 27 λ. Figure 6 shows that when all elements are working, a pattern with a main lobe width of 3.62° and a peak side lobe level of −13.19 dB is generated. The minimum power of each array element is 0.001 (W), and the maximum power is 10 (W).

According to expert experience and some experimental data, the interval of the number of excitation elements is determined by the bell-shaped fuzzy random variable $\xi \left(\omega \right)$, which is expressed as follows

(33) $$\begin{array}{c} \xi ={\left(1+{\left|\frac{x-w}{a_{1}}\right|}^{2b_{{}^{1}}}\right)}^{-1} \end{array}$$

where $a_{1}=4$, $b_{1}=4$. $N\left(\mu ,\sigma^{2}\right)$ refers to the normal distribution with mean μ and variance $\sigma^{2}$. The confidence level is γ=0.8, $\delta =0.8,\beta_{1}=0.8,$ and $\beta_{2}=0.8$.

The hybrid intelligent algorithm is used to solve Equation (20). The expected main lobe width is 3.6°, and the optimal solution is obtained when the normalized peak side lobe level is as small as possible.

**Figure 7:**
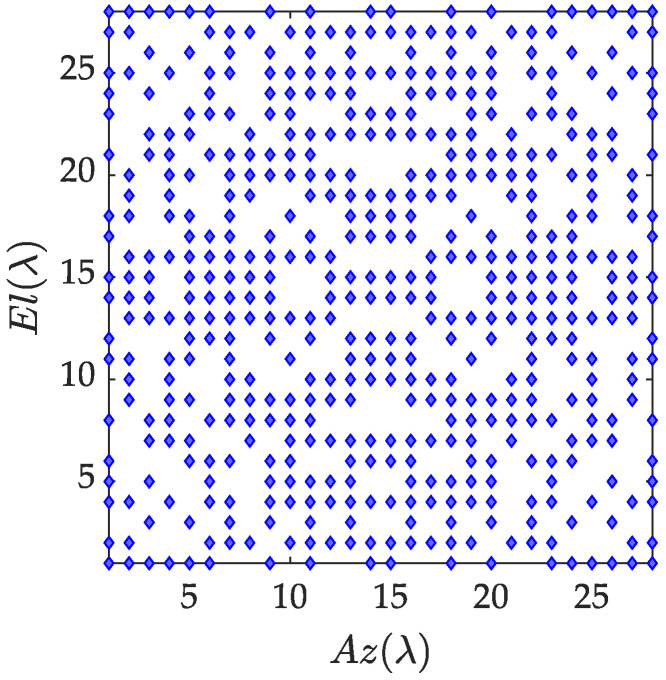
Distribution of array elements after optimization.

**Figure 8:**
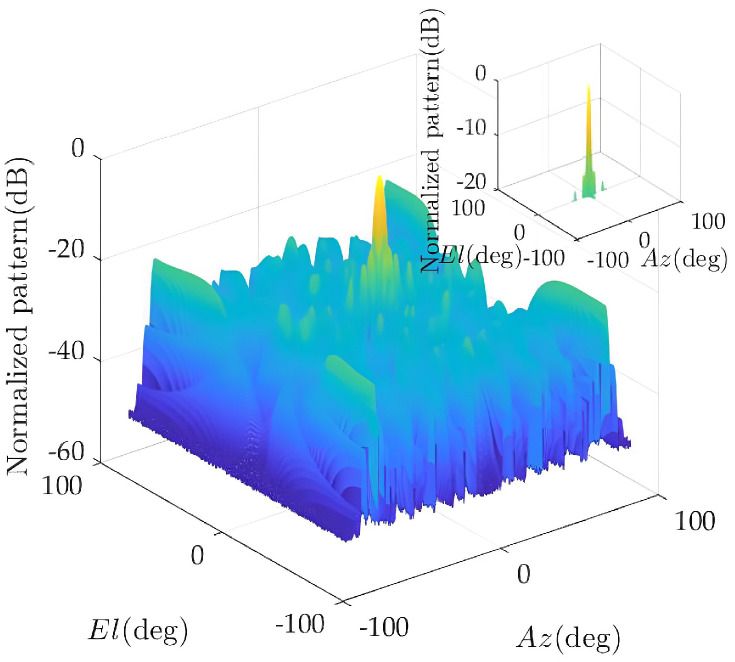
Pattern of the antenna array after optimization.

The element distribution and composite pattern of the optimized solution are given below. Figure 7 shows the element distribution of 504 elements. Figure 8 shows the pattern synthesis results: the main lobe width is 3.65°, and the normalized peak side lobe level is −14.5 dB. Compared with the case where all antenna elements are working, the number of elements in a working state is greatly reduced under the constraint of ensuring the pattern performance.

### 6.3. Minimize Schleher Intercept Factor

**Figure 9:**
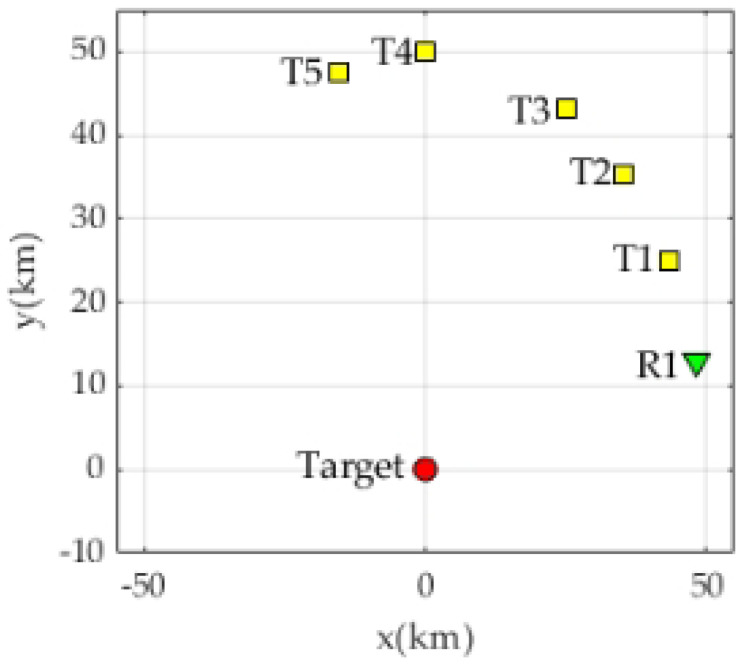
Radar target distribution situation.

The simulation in this section adopts the distributed radar system layout $\left(5\times 1\;\left(M=5,N=1\right)\right)$, as shown in Figure 9. The distance between radar and target is equal, set as $D_{mT}=D_{nT}=50\;\mathrm{km}\;\left(m=1,2,3,4,5,n=1\right)$.

**Figure 10:**
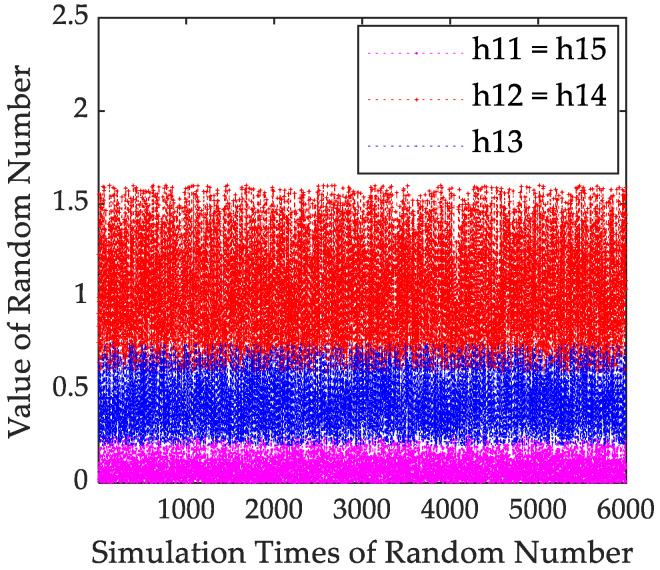
RCS model.

Considering that the RCS of each transceiver path can be modelled as a random variable subject to the Swerling I distribution, the RCS of five different transceiver paths is ${\left|h_{1}\right|}^{2}$. In order to make the theoretical analysis results more consistent with the actual situation, the RCS distribution interval shall be properly truncated. The truncated RCS is shown in Figure 10.

Given the MSE $\eta_{\max}$ of the target location for the case of uniform power distribution $p_{tu}=p_{u}{\left[1,1,\cdots ,1\right]}_{M\times 1}^{T}$, where $p_{u}$ is

(34) $$\begin{array}{c} p_{u}=\left(\frac{1}{\eta_{\max}}\right)\frac{1^{T}b_{e}}{1^{T}A_{e}1} \end{array}$$

Define $p_{uTotal}=p_{u}\cdot M$ to represent the total transmit power under the uniform distribution. According to Equation (34), $p_{u}$ is a function of the RCS random variable, so it is still a random variable. In Appendix C, this paper gives a method to calculate digital features using the CDF in Equation (34).

In order to analyze the impact of the JA scheme proposed in this paper on the performance of radar network LPI, Table 2 compares the power performance of four power control algorithms under different confidence levels. The four algorithms are as follows: (1) the proposed algorithm, (2) the fixed power radar assignment (FPRA) algorithm, (3) the uniform power assignment (upa) algorithm, and (4) the adaptive noncooperative power control (ANCPC) algorithm [35]. It can be seen from Table 3 that at the same confidence level when the algorithm proposed in this paper is used for localization, the total power of the radar network to all targets is the least, which is far lower than that of the FPRA algorithm and the UPA algorithm. The ANCPC algorithm consumes more power than the algorithm proposed in this paper because each participant maximizes its utility function selfishly and rationally.

The layout shown in Figure 9 eliminates the influence of radar range on power allocation results. According to the target prior information obtained from the collective awareness and situation sharing among radars, the hybrid intelligent algorithm and local optimal iterative algorithm are used to solve the CCP problem of Equation (29). The power optimization results are compared with the uniform power allocation results. The system’s total power is $P_{mon}^{\max}=p_{Total}=400\;\left(\mathrm{kW}\right)$. The simulation results are shown in Table 3.

In order to analyze the influence of different confidence levels on the distribution results, this paper sets the confidence levels α as 0.99, 0.95, 0.9, and 0.85, respectively. $p_{uTotal}$ represents the transmission power of each radar when it is evenly distributed under the same chance constraint. $E\left[p_{uTotal}\right]$ is the expected value of uniform transmission power $p_{uTotal}$. $p_{optTotal}^{*}$ is the optimal transmission power according to the power allocation algorithm given in Section 5. The ratio of poptTotal*puTotal reflects the advantage and disadvantages of optimal power compared with uniform power distribution; $\alpha_{net}^{*}$ is a low interception factor for optimal power allocation; $p_{t\min}^{*}$ represents the power allocation of each transmitter during optimal allocation. Table 2 shows that the algorithm in this paper can save roughly 50% of the power resources. The higher the confidence level, the fewer power resources saved, and the higher the low interception factor will be. Intuitively, when the total power consumed is more, the low interception factor is also greater, the probability of not meeting the constraint conditions is less, and the confidence level is higher. It can be seen from the allocation results that more power is allocated to Transmitters 2 and 4 because they have better RCS characteristics than Transmitters 1, 2 and 3.

## 7. Conclusions

For a DRNLS, a joint aperture and power allocation scheme for a radar network localization system based on a Low Probability of Interception optimization is proposed. In order to improve the low interception performance, the RAARM-FRCCP model is established according to the uncertainty of the number and distribution of elements in the working state. Considering the randomness of the target RCS, a CCP is introduced to balance the tracking performance and power resources at a given confidence level. Based on obtaining the minimum number of elements, the MSIF-RCCP model is constructed to minimize the Schleicher interception factor of the system. To meet the system tracking performance requirements, we adjust the transmission power of each radar to improve the low interception performance of the radar network system. Finally, FRS-GA solves the Chance Constrained Programmin model of aperture; RS-GA and LSMIA algorithms are used to solve the nonconvex, nonlinear, constrained, low probability Chance Constrained Programmin model. The simulation results show that the uniform power distribution of RCS with randomness is not necessarily the optimal scheme. Under the condition of satisfying the positioning performance and peak side lobe level, the required number of arrays and power will be reduced to a certain extent. The reduction range between different confidence levels will differ so that the DRNLS has better low interception performance.

The advantage of the scheme proposed in this paper is that it considers the optimal allocation of aperture and power of a radar network localization system under LPI. However, with the increasing performance of hardware processing chips and the gradual development of radar technology, computer resources are no longer the bottleneck of radar development. The disadvantage of the proposed scheme is that it cannot independently configure resources according to the battlefield environment and situation, so it is necessary to design intelligent radar aperture and power allocation algorithms. In the future, we will examine the deep learning technique [36,37,38] for the radar network localization system.

## Figures and Tables

**Table 1:** Probability of interception in different situations.

	${\left.T/\tau \right.}^{a}$	1	2	5	10	20
$P_{\mathrm{fa}}$						
15.00%		15.00%	27.75%	55.60%	80.31%	99.90%
30.00%		30.00%	51.00%	83.20%	97.18%	99.90%

*^a^*$\left.T/\tau \right.$ is the ratio of the total radar scan time to the time the main lobe is intercepted. For example, when $\left.T/i\right.$ = 1, it means that only the main lobe of the radar is intercepted, and it is not intercepted at other times; if $\left.T/i\right.$ = 20, it means that there are 20 intercepted times of the main lobe of the radar is intercepted in total in one scan period, including the case where the main lobe is intercepted only once.

**Table 2:** Comparison of total power of radar network.

	α	0.99	0.95	0.90	0.85
Algorithm					
Proposed Algorithm		85	79	77	73
ANCPC		86	83	78	74
UPA		194	173	160	149
FPRA		200	200	200	200

**Table 3:** Comparison of optimization results of power allocation algorithms.

α	0.99	0.95	0.90	0.85
$p_{uTotal}$	194	173	160	149
$E[p_{uTotal}]$	55	55	55	55
$p_{optTotal}^{*}$	85	79	77	73
$\left.p_{optTotal}^{*}/p_{uTotal}\right.$	0.43	0.46	0.48	0.49
$\alpha_{net}^{*}$	0.85	0.79	0.77	0.73
$p_{t\min}^{*}$	$\left[\begin{array}{c} 1\\ 41\\ 1\\ 41\\ 1 \end{array}\right]$	$\left[\begin{array}{c} 1\\ 38\\ 1\\ 38\\ 1 \end{array}\right]$	$\left[\begin{array}{c} 1\\ 37\\ 1\\ 37\\ 1 \end{array}\right]$	$\left[\begin{array}{c} 1\\ 35\\ 1\\ 35\\ 1 \end{array}\right]$

## Data Availability

Not applicable.

## Abbreviations

The following abbreviations are used in this manuscript:

LPI	Low Probability of Intercept
DRNLS	Distributed Radar Network Localization System

## Appendix A

Without losing generality, let the target RCS obeys the exponential distribution with parameter λ, that is, $h_{m,n}∼Exp\left(\lambda \right)$, then there is the following theorem.

 **Theorem A1.**
*When $h_{m,n}∼Exp\left(\lambda \right)$ and $w_{m,n}\left(t\right)∼N\left(0,\sigma_{w}^{2}\right)$, the probability density function of the baseband $r_{m,n}\left(t\right)$ is*

(A1)
$$\begin{array}{c} f_{r_{m,n}\left(t\right)}\left(z\right)=\frac{\lambda }{2a}\exp\left[\frac{\lambda^{2}-4a\lambda z+4a\lambda \mu }{8a^{2}\sigma^{2}}\right]\mathrm{erfc}\left(\frac{\lambda -2az}{2\sqrt{2}a\sigma }\right) \end{array}$$

*where $a=\sqrt{\alpha_{m,n}p_{m_{tx}}}s_{m}\left(t-\tau_{m,n}\right)$. Given the parameter vector $u={\left[x,y,h^{T}\right]}^{T}$, the conditional joint probability density function of the observation vector r is*

(A2)
$$\begin{array}{c} f\left(r|u\right)={\left(\frac{\lambda }{2a}\right)}^{MN}\exp\left[\frac{MN\lambda \left(\lambda -4az+4a\mu \right)}{8a^{2}\sigma^{2}}\right]\prod_{n=1}^{N}\prod_{m=1}^{M}\mathrm{erfc}\left(\frac{\lambda -2az}{2\sqrt{2}a\sigma }\right) \end{array}$$

*where erfc(·) is a complementary error function in the form of $erfc\left(x\right)=\left(\left.2/\sqrt{\pi }\right.\right)\int_{x}^{+\infty }e^{-t^{2}}dt$.*

**Proof.** For the convenience of discussion, $\sqrt{\alpha_{m,n}p_{m_{tx}}}s_{m}\left(t-\tau_{m,n}\right)$ in Equation (4) is represented by *a*, then the baseband $r_{m,n}\left(t\right)$ can be expressed as

(A3) $$\begin{array}{c} r_{m,n}\left(t\right)=ah_{m,n}+w_{m,n}\left(t\right) \end{array}$$

Since the random variables $h_{m,n}$ and $w_{m,n}\left(t\right)$ are independent of each other, the following equation can be obtained according to the convolution formula

(A4) $$\begin{array}{cc} f_{r_{m,n}\left(t\right)}\left(z\right) & =\int_{0}^{+\infty }f_{h_{m,n}\left(t\right)}\left(x\right)f_{w_{m,n}\left(t\right)}\left(z-ax\right)dx\\ \; & =\frac{\lambda }{\sqrt{2\pi }\sigma }\int_{0}^{+\infty }\exp\left[-\lambda x-\frac{{\left(z-ax\right)}^{2}}{2\sigma_{w}^{2}}\right]dx\\ \; & =\frac{\lambda }{\sqrt{2\pi }\sigma }\exp\left[\frac{\lambda \left(\lambda -4az+4a\mu \right)}{8a^{2}\sigma^{2}}\right]\int_{0}^{+\infty }\exp\left[-{\left(\frac{4a^{2}x+\lambda -2az}{2\sqrt{2}a\sigma }\right)}^{2}\right]dx \end{array}$$

Let $t=\left.\left(4a^{2}x+\lambda -2az\right)/\left(2\sqrt{2}a\sigma \right)\right.$, and combine with complementary error function, Equation (A4) can be simplified as

(A5) $$\begin{array}{cc} f_{r_{m,n}\left(t\right)}\left(z\right) & =\frac{\lambda }{2a}\exp\left[\frac{\lambda \left(\lambda -4az+4a\mu \right)}{8a^{2}\sigma^{2}}\right]\left[\frac{2}{\sqrt{\pi }}\int_{\frac{\lambda -2az}{2\sqrt{2}a\sigma }}^{+\infty }e^{-t^{2}}dt\right]\\ \; & =\frac{\lambda }{2a}\exp\left[\frac{\lambda \left(\lambda -4az+4a\mu \right)}{8a^{2}\sigma^{2}}\right]\mathrm{erfc}\left(\frac{\lambda -2az}{2\sqrt{2}a\sigma }\right) \end{array}$$

According to the conditions given in the text, different basebands are independent of each other. Then, given the parameter vector u, the conditional joint probability density of the observation vector r is

(A6) $$\begin{array}{cc} f\left(r|u\right) & =\prod_{n=1}^{N}\prod_{m=1}^{M}f_{r_{m,n}\left(t\right)}\left(z\right)\\ \; & =\prod_{n=1}^{N}\prod_{m=1}^{M}\frac{\lambda }{2a}\exp\left[\frac{\lambda \left(\lambda -4az+4a\mu \right)}{8a^{2}\sigma^{2}}\right]\mathrm{erfc}\left(\frac{\lambda -2az}{2\sqrt{2}a\sigma }\right)\\ \; & ={\left(\frac{\lambda }{2a}\right)}^{MN}\exp\left[\frac{MN\lambda \left(\lambda -4az+4a\mu \right)}{8a^{2}\sigma^{2}}\right]\prod_{n=1}^{N}\prod_{m=1}^{M}\mathrm{erfc}\left(\frac{\lambda -2az}{2\sqrt{2}a\sigma }\right) \end{array}$$

Equations (A5) and (A6) are the conclusions in this theorem. □

## Appendix B

From Section 5, it can be seen that the RCS obeys the exponential distribution with the domain of [0,+∞). When the parameter λ is given, it is improbable that RCS will take a value far away from the expectation $\left.1/\lambda \right.$, which is also a small probability event in reality. Therefore, this paper will truncate the exponential distribution that RCS obeys appropriately for subsequent analysis.

Let $h_{m,n}∼Exp\left(\lambda \right)$, then the probability density function of $h_{m,n}$ is

(A7) $$\begin{array}{c} f\left(x\right)=\left\{\begin{array}{cc} \lambda e^{-\lambda x}, & x⩾0,\\ 0, & x<0. \end{array}\right. \end{array}$$

If the range of *x* is truncated within the interval [a,b], the truncated probability density function can be set as $\tilde{f}\left(x\right)=Af\left(x\right)$. According to the normalization of distribution, the following equation can be obtained

(A8) $$\begin{array}{c} \int_{a}^{b}Af\left(x\right)dx=1\Rightarrow A\int_{a}^{b}\lambda e^{-\lambda x}dx=1\Rightarrow A=e^{-\lambda a}-e^{-\lambda b} \end{array}$$

Therefore, the probability density function of exponential distribution with truncation interval [a,b] is

(A9) $$\begin{array}{c} \tilde{f}\left(x\right)=\frac{\lambda e^{-\lambda x}}{e^{-\lambda a}-e^{-\lambda b}} \end{array}$$

From the relationship between cumulative distribution function and probability density function, the cumulative distribution function of exponential distribution with truncation interval of [a,b] is

(A10) $$\begin{array}{c} \tilde{F}\left(x\right)=\left\{\begin{array}{cc} 0, & x<a,\\ \frac{e^{-\lambda a}-e^{-\lambda x}}{e^{-\lambda a}-e^{-\lambda b}}, & a⩾x<b,\\ 1, & x⩾b. \end{array}\right. \end{array}$$

The correctness of Appendix B has been verified in Section 6 of the text.

## Appendix C

For the convenience of discussion, Let $Y=|h^{2}|$ and g(Y) be a function of the random variable Y. According to the analysis in the first paragraph of Section 6, the following theorem is given.

 **Theorem A2.**
*Let the probability density function, cumulative distribution function, and domain of random variable g(Y) be $\tilde{f}\left(y\right)$, $\tilde{F}\left(y\right)$, and [0,+∞), respectively, then the expected value of g(Y) can be calculated according to the following equation*

(A11)
$$\begin{array}{c} E\left[g\left(Y\right)\right]=\int_{0}^{+\infty }\left[1-\tilde{F}\left(y\right)\right]dy \end{array}$$

**Proof.** Combined with the conditions given in this theorem and the definition of expectation, the expectation of random variable g(Y) is

(A12) $$\begin{array}{c} E\left[g\left(Y\right)\right]=\int_{0}^{+\infty }y\tilde{f}\left(y\right)dy \end{array}$$

Substitute $y=\int_{0}^{y}1dz$ into Equation (A12) and sort it out according to the property of successive integration, then the following equation can be obtained

(A13) $$\begin{array}{cc} E\left[g\left(Y\right)\right] & =\int_{0}^{+\infty }\left[\int_{0}^{y}1dz\right]\tilde{f}\left(y\right)dy\\ \; & =\int_{0}^{+\infty }\left[\int_{0}^{y}\tilde{f}\left(y\right)dz\right]dy\\ \; & =\int_{z}^{+\infty }\left[\int_{0}^{y}\tilde{f}\left(y\right)dz\right]dy\\ \; & =\int_{0}^{+\infty }\left[\int_{z}^{y}\tilde{f}\left(y\right)dy\right]dz\\ \; & =\int_{0}^{+\infty }\left[1-\tilde{F}\left(z\right)\right]dz \end{array}$$

Since the integral result is independent of the variable symbol, replace *z* with *y*, and Equation (A11) holds. □

Theorem A2 uses the cumulative distribution function instead of the probability density function to calculate the expected value, ensuring that the correct results can be obtained using random simulation technology to calculate the expected value of continuous random variables.
